# Management of Fever in Postpneumococcal Vaccine Era: Comparison of Management Practices by Pediatric Emergency Medicine and General Emergency Medicine Physicians

**DOI:** 10.1155/2014/702053

**Published:** 2014-06-01

**Authors:** Hnin Khine, David L. Goldman, Jeffrey R. Avner

**Affiliations:** ^1^Division of Pediatric Emergency Medicine, Children's Hospital at Montefiore, 3415 Bainbridge Avenue, Bronx, NY 10467, USA; ^2^Department of Pediatrics, Albert Einstein College of Medicine, Bronx, NY 10467, USA; ^3^Division of Pediatric Infectious Diseases, Children's Hospital at Montefiore, Bronx, NY 10467, USA

## Abstract

*Background*. The primary objective of this study was to compare management practices of general emergency physicians (GEMPs) and pediatric emergency medicine physicians (PEMPs) for well-appearing young febrile children. *Methods*. We retrospectively reviewed the charts of well-appearing febrile children aged 3–36 months who presented to a large urban children's hospital (PED), staffed by PEMPs, or a large urban general emergency department (GED), staffed by GEMPs. Demographics, immunization status, laboratory tests ordered, antibiotic usage, and final diagnoses were collected. *Results*. 224 cases from the PED and 237 cases from the GED were reviewed. Children seen by PEMPs had significantly less CXRs (23 (10.3%) versus 51 (21.5%), *P* = 0.001) and more rapid viral testing done (102 (45%) versus 40 (17%), *P* < 0.0001). A diagnosis of a viral infection was more common in the PED, while a diagnosis of bacterial infection (including otitis media) was more common in the GED. More GED patients were prescribed antibiotics (41% versus 27%, *P* = 0.002), while more PED patients were treated with oseltamivir (6.7% versus 0.4%, *P* < 0.001). *Conclusions*. Our findings identify important differences in the care of the young, well-appearing febrile child by PEMPs and GEMPs and highlight the need for standardization of care.

## 1. Introduction

Physicians with varying amounts of pediatric clinical experience manage children seeking emergency care. Because of differences in training and work exposures, the management of ill and injured pediatric patients in the emergency department can vary greatly between pediatric emergency physicians (PEMPs) and general emergency medicine physicians (GEMPs). Prior studies have shown important differences in the management of pediatric patients by GEMPs and PEMPs in a variety of clinical situations including, resuscitation, fracture management, and resource utilization [1–4].

Fever is one of the most common chief complaints of children presenting to the emergency department. This includes otherwise well-appearing children aged 3–36 months, who are at risk for occult bacteremia. In the 1980s and early 1990s, the standard of care for these children included laboratory testing (complete blood count and blood culture) and empiric antibiotic treatment. Practice guidelines from 1993 recommended that children of 3 to 36 months of age with fever of 39.0°C or higher and whose WBC count was 15,000/mm^3^ or more should have a blood culture performed and be treated with antibiotics pending culture results [5]. With the advent improved conjugated pneumococcal and* Haemophilus* vaccines, the incidence of occult bacteremia in vaccinated, well-appearing febrile children has dramatically decreased and is now estimated to be 0.25–0.7% [6]. Consequently, the risk and cost of routine testing and treating young febrile children for bacteremia likely exceed their potential benefits. A recent review suggests that the only laboratory tests necessary in the evaluation of fever >39.0°C in young children are a urinalysis and urine culture (for circumcised males <6 months of age and uncircumcised males and females <24 months of age) [7].

There have been no recent comparisons of PEMPs with GEMPs in their evaluation and management of well-appearing young children with fever in the postpneumococcal vaccine era. A questionnaire performed by Maldonado et al. found that while PEMPs and GEMPs agreed on level of triage for most pediatric scenarios, GEMPs triaged children with fever at a significantly higher level of triage than PEMPs [8]. Despite the availability of specialized care for children in designated children's centers, the majority of children are cared for in general emergency departments. Differences in care among these sites can have important medical and economic implications. We hypothesized that GEMPs are likely to perform more tests and more likely to prescribe antibiotics when compared with PEMPs.

## 2. Methods

We performed a chart review of cases of well-appearing febrile children aged 3–36 months who presented to either a large urban children's hospital emergency department (PED) or a large urban general emergency department (GED) from September 1, 2009, to December 31, 2009. The children's hospital was located in the urban setting with an annual census of 60,000 per year. The GED was located 5 miles east of the children's hospital and has an annual census of 72,000 per year, 18% of whom are children. The children's hospital is a teaching hospital with residency and fellowship program while the general ED is staffed by general emergency medicine attending physicians and physician assistants. Pediatric patients from both hospitals were admitted to the children's hospital.

Charts were identified through the ED charting system, which is used by both hospitals. Charts were reviewed if the patient was between 3 and 36 months with the triage chief complaint of fever. The investigators manually reviewed all the eligible patients' records from the electronically scanned charts. Patients were excluded if they were admitted or had underlying conditions that predispose to infection, including an indwelling catheter (venous, bladder, or ventriculoperitoneal shunt), sickle cell disease, immunocompromising condition, cancer, or other chronic illness.

Demographic data were extracted from the ED charts of eligible patients using a standardized data collection sheet. In addition, the following information was collected: maximal height of fever (both historical and triage values), immunization status, number and types of laboratory tests ordered, use of antibiotics (type of antibiotic and route of administration), procedures performed, and final diagnosis. Final diagnoses were classified into either viral or bacterial based on the diagnosis and the use of antibiotics.

### 2.1. Statistics

#### 2.1.1. Power Calculations

The primary outcome variable was the number of children receiving blood tests. Secondary outcomes included the number of tests performed, the use of empiric antibiotics, and the route of administration of antibiotics. We estimated that about 2.5% of eligible patients at PED received blood testing. In order to detect a 3-fold increase in number of eligible patients receiving blood tests in GED with an alpha error of 0.5 and a statistical power of 0.8, a sample size of 234 in each group was required. During the study period, an average of 81 children aged 3–36 months with fever were seen in the GED monthly, while 411 were seen in the PED monthly. In order to collect patients over the same 4-month interval, we reviewed every 6th patient in the PED group. The investigators manually reviewed every 6th chart of patients, aged 3–36 months with fever. If a patient met the exclusion criteria, the chart of the patient that preceded the eligible patient was reviewed. However, if the preceding patient also met the exclusion criteria, then the patient was counted as excluded.

### 2.2. Data Analysis

Categorical data such as sex and testing status were compared using chi-square or Fisher's exact test. Quantitative data was tested for normality using the Shapiro-Wilk normality test. Nonnormally distributed data was compared using the Mann-Whitney *U* test. Normally distributed data was compared using the Student's *t*-test. *P* values < 0.05 were considered significant. All statistics were done with GraphPad InStat software (San Diego, CA). This study was approved by the hospital's committee on clinical investigations.

## 3. Results

**Figure 1:**
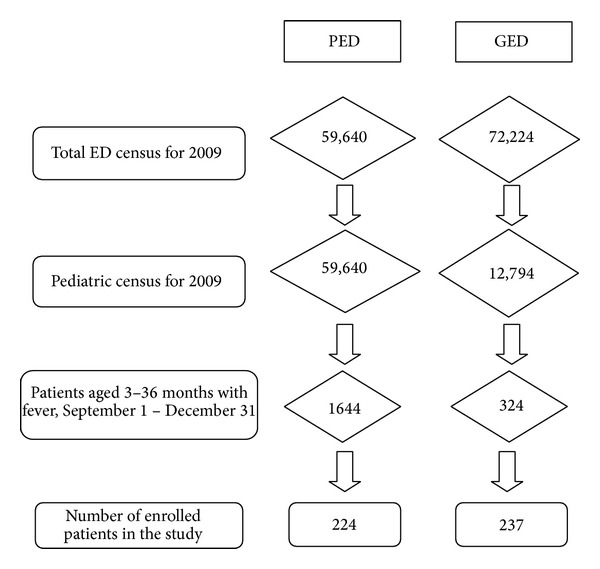
Hospital ED census and enrollment numbers are given for the study period, September 1, 2009, to December 31, 2009.

For 2009, the annual census for the PED was 59,640 patients. In contrast, the annual census for the GED was 72,224 patients of which 12,794 (18%) were pediatric patients. The PED was staffed by 32 providers, while the GED was staffed by 36 providers. During the study period, from the PED, 1644 visits of patients aged 3–36 months with a chief complaint of fever or febrile seizure were identified, of which every 6th chart (274) was reviewed. Fifty of these cases were excluded based on criteria, as outlined above. From the GED, 324 charts were identified and 87 excluded based on criteria. A total of 461 charts (224 from PED and 237 from GED) were reviewed for this study (Figure 1). Demographic data are shown in Table 1. The two groups were similar in terms of age, sex, and insurance status; however, the patients in PED group had a higher temperature, both by history and by triage. Documentation of immunization status by either nurse or physician was more common in PED cases (222/224, 98%) than in GED cases (200/237, 84%, *P* < 0.001).

The frequency of laboratory testing by PEMPs and GEMPs is shown in Table 2. Both PEMPs and GEMPs infrequently obtained complete blood counts and blood cultures. Furthermore, both PEMPs and GEMPs ordered similar numbers of urine cultures. However, GEMPs ordered more chest radiographs while PEMPs ordered more viral testing. The discharge diagnoses of the study patients are listed in Table 3. Overall, the most common diagnosis made by both PEMPs and GEMPs was viral illness/viral syndrome. However, more patients treated by GEMPs received a diagnosis with a bacterial cause compared to those patients treated by PEMPs. GEMPs diagnosed more acute otitis media as well as pharyngitis than PEMPs (Table 3). Overall, GEMPs prescribed more antibiotics than PEMPs (97/237 (41%) versus 61/224 (27%), *P* = 0.002). Consistent with more viral testing, PEMPs prescribed more antivirals (primarily oseltamivir) than GEMPs (7% versus 0.4%, *P* < 0.001).

## 4. Discussion

The majority of children who seek emergency care in the United States are treated in hospitals staffed by GEMPs. Children under the age of 18 comprised 22.1% of all hospital emergency department visits. Nonetheless, the annual number of pediatric patients seen in most general hospital EDs is relatively small. The National Health Statistic Report from 2012 noted that 4000 out of 4800 hospital emergency departments see less than 10,000 pediatric patients per year [9]. The relatively limited exposure to pediatric emergencies during training and practice can make the managing of pediatric emergencies especially challenging for the GEMP.

A survey conducted in 2001 to emergency physicians regarding their management of febrile young children reported a very high rate of invasive testing with 69% of correspondents choosing to perform complete blood count and 46% choosing to perform blood cultures [10]. In contrast, we found that the overall rate of invasive testing was low for both GEMPs and PEMPS. The hypothesis that GEMPs rely more heavily on invasive testing to assess young febrile children of 3–36 months of age was not supported by our findings. This minimalistic approach is consistent with recent recommendations that recommend the elimination of routine invasive testing for well-appearing young febrile children based on very low rates of occult bacteremia (<0.5%) among vaccinated children [11, 12].

However, there were important differences in the care provided in the GED and the PED. Firstly, the documentation of immunization status was significantly greater in the PED when compared with the GED. Immunization status is an important consideration in the evaluation of the febrile infant since unvaccinated infants are at increased risk for pneumococcal bacteremia [13]. In addition, we found significantly higher rates of antibiotic prescription and diagnoses of AOM by GEMPs when compared with PEMPs. These findings are similar to those of Isaacman et al. who found that GEMPs diagnosed more bacterial focal infections, mainly otitis media, and prescribed more antibiotics than PEMPS when caring for children with fever [10]. Overuse of antibiotics has important ramifications including the emergence of resistant organisms, like penicillin resistant pneumococci and highly resistant gram-negative organisms. Overuse of antibiotics is also associated with increased cost and adverse effects including anaphylaxis and* C. difficile *colitis. In attempt to limit antibiotic exposure in the pediatric community, the AAP recommends an initial observation for children over 23 months with acute otitis media [14].

We also found that performance of CXR in the evaluation of young febrile child was greater among GEMPs. Again these findings are consistent with those of Isaacman et al. [10]. Nonetheless, the rate of CXR used by GEMPs was significantly lower than that reported in the Isaacmen study (21% versus 40%). Previous studies have noted a wide variation in the utilization of chest radiography in the evaluation of febrile children in PEDs [15]. Guidelines for the use of chest radiographs in the evaluation of children older than 3 months with fever are not precise. The 2003 ACEP Clinical Policies Committee recommends (level C) that a chest radiograph is usually not indicated in febrile children aged older than 3 months with temperature less than 39°C (<102.2°F) without clinical evidence of acute pulmonary disease [16]. Not surprisingly, high chest radiography rates have been associated with increased antibiotic usage [17].

The high rate of viral testing by PEMPs in this study was likely affected by the timing of this study relative to H1N1 outbreak. Children under 2 years of age were considered to be in the high-risk group for complication from influenza and thus were screened for influenza including H1N1. This pattern of practice was reflected in the higher percent of use of antiviral agents among PEMPs. The cost effectiveness of this practice is yet to be determined.

There were several limitations of the study. This was a retrospective study and therefore we cannot comment on the decision making process of physicians. We believe that the two groups of patients that were included in the study were similar in terms of their health care need since the 2 hospitals are geographically located in close proximity. Children presenting to PED may be sicker since more patients with complex health history will seek the specialized care at children's hospital. However, in this study, we only included healthy, well-appearing children; thus we believe that their exposure to pathogens and external factors should be similar. We relied on the clinician assessment of well-appearing child as an inclusion criterion. This may have minimized the potential difference in the test performing behavior between the 2 groups since we are not able to determine the ability of physician's assessment of a healthy child. A child who may have been admitted to the hospital by GEMPs may not have been considered ill by PEMPs. We also included limited number of hospital sites, which may affect the generalizability of our findings. Nonetheless, we note that our findings are consistent with earlier studies regarding differences in the treatment of children by PEMPs versus GEMPs [1, 10].

## 5. Conclusions

Our studies indicate that the rate of invasive testing for well-appearing young febrile children at risk for occult bacteremia is low in EDs staffed by PEMPs and GEMPS. Nonetheless, the use of antibiotics and X-rays by GEMPs remains high when compared with PEMPs. In contrast, both the diagnosis of influenza and oseltamivir use are significantly more common with PEMPS. These findings are consistent with those of previous studies and highlight the differences in care received by febrile children cared for by PEMPs and GEMPs.

## Figures and Tables

**Table 1:** Demographics.

	PED	GED	*P* value
Age (mos)	16.6 ± 9.1	15.2 ± 8.7	NS
Male (%)	56%	49%	NS
*T*_max⁡_ by hx (°F)	102.7 ± 1.4	102.2 ± 1.4	0.01
Triage temperature (°F)	101.8 ± 1.9	101.3 ± 1.9	0.03
Immunization documented (%)	98%	84%	<0.001
State assistance insurance/self-pay (%)	61%	56%	NS

**Table 2:** Laboratory test performed.

	PED (*n* = 224)	GED (*n* = 237)	*P* value
Diagnostic studies			
CBC (%)	8 (4)	9 (4)	NS
BCX (%)	8 (4)	7 (3)	NS
UCX (%)	20 (9)	11 (5)	0.09
CXR (%)	23 (10)	51 (22)	0.001
VIRAL testing (%)	102 (46)	40 (17)	<0.001

**Table 3:** Discharge diagnoses and prescription patterns.

	PED (*n* = 224)	GED (*n* = 237)	*P* value
	Diagnosis		
Viral (total)	**163**	**140**	**0.0016**
Viral syndrome	103	122	NS
Influenza	16	0	<0.0001
Bronchiolitis	15	8	NS
AGE*	13	5	0.053
Fever	9	1	0.0091
Stomatitis/herpangina	5	2	NS
Croup	2	2	NS
Bacterial (total)	**61**	**97**	**0.0017**
Otitis media	50 (22)	77 (32)	0.016
UTI/pyelonephritis	3	1	NS
Pneumonia	3	3	NS
Sinusitis	4	1	NS
Pharyngitis	0	10	0.0018
Lymphadenitis	1	0	NS
Bronchitis	0	3	NS
Conjunctivitis	0	2	NS

	Prescriptions		
Antibiotics	61 (27)	97 (41)	0.002
Antiviral	15 (7)	1 (0)	<0.001

*Refers to acute gastroenteritis.
